# Development of Dual Enzymatic–Ionic Crosslinked Alginate–Gelatin Films Enriched with *Pelargonium sidoides* Extract for Active Packaging and Anthocyanin Preservation in Flame Seedless Grapes

**DOI:** 10.3390/polym18192356

**Published:** 2026-09-27

**Authors:** Renata Dobrucka, Marcin Szymański

**Affiliations:** 1 Department of Non-Food Products Quality and Packaging Development, Institute of Quality Science, Poznan University of Economics and Business, al. Niepodległości 10, 61-875 Poznan, Poland; 2 Center for Advanced Technologies, Adam Mickiewicz University in Poznan, ul. Uniwersytetu Poznańskiego 10, 61-614 Poznan, Poland

**Keywords:** protein–polysaccharide films, dual cross-linking, Ca^2+^ ions, transglutaminase

## Abstract

Active biodegradable packaging materials capable of preserving fruit quality and reducing oxidative processes are promising alternatives to conventional petroleum-based plastics. In this study, alginate–gelatin composite films were developed using a dual crosslinking strategy combining microbial transglutaminase-mediated enzymatic crosslinking with Ca^2+^-induced ionic gelation. The films were then enriched with *Pelargonium sidoides* root extract as a natural antioxidant. The combined crosslinking approach resulted in a more compact protein–polysaccharide network, which was reflected in increased film density and reduced swelling. The highest density was observed for film 1.0F (ρ = 1.2004 ± 0.0151 g/cm^3^), while films containing Ca^2+^ showed lower swelling values, with SI_60_ decreasing from 7655 ± 70 for 0F to 5853 ± 49 for 0Ca^2+^F. The addition of the extract promoted additional interactions within the polymer matrix and affected the swelling and barrier properties of the films. The WVTR values were 36.1 ± 0.9 g/m^2^·24 h for 1.0F and 49.8 ± 1.5 g/m^2^·24 h for 1.0Ca^2+^F. The incorporation of *P. sidoides* extract also significantly increased the antioxidant activity of the films, particularly at higher extract concentrations. Furthermore, films containing Ca^2+^ and the extract contributed to the preservation of anthocyanins in Flame Seedless grapes during 14 days of storage, with the anthocyanin content reaching 0.208% ± 0.019 for the 1.0Ca^2+^F treatment. Overall, the results indicate that dual-crosslinked alginate–gelatin films enriched with *P. sidoides* extract have favorable physicochemical and functional properties and may be suitable for the development of sustainable active packaging materials for anthocyanin-rich fruits.

## 1. Introduction

The global fruit and vegetable market continues to expand, driven by increasing consumer awareness of the relationship between diet and health and by the growing demand for fresh, minimally processed, and naturally derived foods. Packaging of fresh fruits represents a major technological challenge because of the large volume of products requiring protection and the need to maintain quality throughout storage and distribution. Fresh fruits are highly perishable commodities characterized by intensive metabolic activity and susceptibility to moisture loss, microbial spoilage, and oxidative deterioration. Consequently, large quantities of packaging materials are required to preserve their quality and extend shelf life. At present, most packaging materials used for fresh produce are petroleum-based plastics. Although these materials exhibit excellent mechanical and barrier properties, their extensive use has raised significant environmental concerns. Moreover, recycling of fruit packaging is often inefficient because the materials are frequently contaminated with organic residues, moisture, and fruit exudates, which reduce recycling efficiency and increase processing costs [1,2,3]. These limitations have stimulated intensive research into biodegradable and sustainable alternatives for food packaging applications.

Biopolymer-based films and coatings have emerged as promising candidates to replace conventional plastics owing to their biodegradability, renewability, and suitability for food-contact applications. Nevertheless, only a limited number of these materials have reached commercial implementation. Their limited industrial application is mainly associated with insufficient mechanical strength, poor barrier properties against water vapor and oxygen, and inadequate functional performance, including antioxidant and antimicrobial activity [4]. Consequently, numerous strategies have been proposed to improve the physicochemical and functional properties of edible biopolymer films and coatings [5,6]. Among these approaches, cross-linking has attracted particular attention because it strengthens polymer networks, enhances mechanical stability, reduces water solubility and swelling, and improves barrier performance. Besides polymer blending, both chemical and enzymatic crosslinking have been extensively investigated as effective approaches for stabilizing polymer matrices [7]. Therefore, it may be hypothesized that combining ionic (Ca^2+^-mediated) and enzymatic (transglutaminase-induced) crosslinking within alginate–gelatin systems can produce films with improved structural organization and enhanced functional properties suitable for active food packaging applications.

Sodium alginate and gelatin were selected as the principal film-forming biopolymers for the development of active packaging materials intended for Flame Seedless grapes. Gelatin, a product of collagen hydrolysis designated as food additive E441, is widely used because of its excellent film-forming ability, biodegradability, edibility, availability, and relatively low cost [8,9]. Owing to these properties, gelatin has found extensive applications in the food, pharmaceutical, cosmetic, and biomedical industries. Furthermore, recent studies have demonstrated that blending gelatin with polysaccharides significantly improves film functionality and structural stability, resulting in materials with enhanced performance characteristics [10]. Sodium alginate is a naturally occurring unbranched copolymer composed of β-D-mannuronic and α-L-guluronic acid residues linked through (1→4) glycosidic bonds and arranged in varying proportions and sequences. It is naturally present as a structural component of brown seaweeds and as a capsular polysaccharide in certain bacterial species [11]. The abundance of carboxyl groups within alginate chains enables interactions with multivalent cations, particularly calcium ions, leading to the formation of ionically crosslinked structures according to the well-established “egg-box” model [12]. Despite these advantages, alginate-based materials generally exhibit limited moisture resistance and relatively poor mechanical properties because of their hydrophilic nature.

To overcome these limitations, both ionic and enzymatic crosslinking were employed to develop structurally reinforced alginate–gelatin films. Calcium ions promote the formation of ionic junction zones within the alginate phase, whereas microbial transglutaminase catalyzes the formation of covalent ε-(γ-glutamyl)lysine isopeptide bonds within the gelatin network, thereby enhancing its structural stability. In addition, electrostatic interactions and hydrogen bonding between alginate and gelatin may further contribute to the formation of a hierarchically organized protein–polysaccharide network with improved physicochemical performance. Enzymatic modification has attracted considerable attention as an efficient, environmentally friendly, and mild strategy for tailoring the properties of protein-based materials [13]. Although transglutaminase has been extensively applied in protein systems, its use in protein–polysaccharide matrices remains comparatively less explored. Previous studies have demonstrated that transglutaminase-mediated crosslinking improves the functional properties of protein blends, including enhanced emulsifying properties in β-lactoglobulin–soy 11S protein systems [14]. Since the pioneering work of Mahmoud and Savello [15], numerous studies have confirmed the effectiveness of transglutaminase as a crosslinking agent for the preparation of biodegradable films and coatings intended for food applications. The combination of Ca^2+^-mediated ionic crosslinking and transglutaminase-induced enzymatic crosslinking enables the formation of a dual-crosslinked network consisting of ionic interactions within the polysaccharide phase and covalent isopeptide bonds within the protein matrix. Such a strategy is expected to generate a more compact and stable polymer architecture with superior physicochemical properties compared with systems crosslinked solely by ionic or enzymatic mechanisms.

An additional innovative aspect of this study is the incorporation of Pelargonium sidoides root extract. *P. sidoides* is recognized as a valuable source of biologically active compounds, including highly oxidized coumarins, flavonoids, hydroxybenzoic acid derivatives, hydroxycinnamic acid derivatives, and oligomeric proanthocyanidins [16,17]. These phytochemicals exhibit pronounced antioxidant activity and may contribute to reducing oxidative deterioration in packaged food products. Moreover, phenolic constituents may interact with the polymer matrix through hydrogen bonding and other intermolecular interactions, further influencing the structural organization and functional performance of the developed films. Therefore, the incorporation of Pelargonium extract into dual-crosslinked alginate–gelatin matrices offers the opportunity to obtain multifunctional active packaging materials with enhanced antioxidant capacity and improved physicochemical properties. Such an approach is particularly relevant for fresh and minimally processed fruits, where limiting oxidation, reducing quality deterioration, maintaining sensory attributes, and preserving anthocyanins are of considerable importance.

The novelty of the present study lies in the development of alginate–gelatin composite films integrating two complementary crosslinking mechanisms, namely Ca^2+^-mediated ionic crosslinking and transglutaminase-induced enzymatic crosslinking, together with the incorporation of Pelargonium sidoides root extract. To the best of our knowledge, studies describing the simultaneous application of these two crosslinking strategies within a single protein–polysaccharide system enriched with a Pelargonium-derived bioactive extract remain limited. Therefore, the objective of this study was to develop and characterize dual-crosslinked alginate–gelatin films containing *Pelargonium sidoides* extract and to evaluate their structural, physicochemical, mechanical, barrier, and antioxidant properties, as well as their effectiveness in preserving anthocyanins in Flame Seedless grapes during storage. It was hypothesized that the combination of dual crosslinking and a plant-derived antioxidant would generate a compact multifunctional polymer network with enhanced physicochemical performance and improved preservation efficacy.

## 2. Materials and Methods

### 2.1. Materials

Low-molecular-weight gelatin (1% *w*/*w*) and sodium alginate (1% *w*/*w*) were used to prepare the film-forming solutions. Gelatin (Type A, from porcine skin, Sigma-Aldrich, St. Louis, MO, USA) and sodium alginate (from brown algae, guluronic acid content approximately 70%, mannuronic acid content approximately 30%, Mw of approximately 100,000–200,000 g/mol; Sigma-Aldrich, St. Louis, MO, USA) were combined at a 2:1 weight ratio. Distilled water (Poland) served as the main solvent, and glycerol (≥99%, Chempur, Piekary Śląskie, Poland) was added at 1% (*w*/*w*) as a plasticizer. Transglutaminase from guinea pig liver (T5398, ≥1.5 units/mg protein), ethanol, and calcium chloride (CaCl_2_, analytical grade) were obtained from Sigma-Aldrich (St. Louis, MO, USA). Hydrochloric acid (HCl, analytical grade), methanol, DPPH (2,2-diphenyl-1-picrylhydrazyl), potassium persulfate (K_2_S_2_O_8_), ABTS, sodium acetate, and potassium chloride were likewise purchased from Sigma-Aldrich. Distilled water was used throughout all experiments. All reagents were of analytical grade and used without further purification.

### 2.2. Preparation of Pelargonium Extract

Dried and finely ground *P. sidoides* roots were extracted using 50% (*v*/*v*) ethanol in water. The extraction was performed under reflux for 2 h on a heating mantle, using a plant material-to-solvent ratio of 1:10 (*w*/*v*). The resulting filtrate was concentrated under reduced pressure using a rotary evaporator at a temperature not exceeding 40 °C to obtain a 50% ethanol extract in water. The extract was then adjusted to a final concentration of 1 g of raw material per mL by controlled removal of ethanol.

In order to standardise the extract, an extract was prepared from 100 g of raw material in accordance with the above methodology and then evaporated to dryness under reduced pressure in an evaporator. The dry matter content was calculated, and the extraction yield was then determined using the formula:

Y[%] = 100 × m_dry extract_/m_material_

m_material_—mass of raw material [g]

m_dry extract_—mass of dry extract [g]

As a result of evaporating the prepared extract to dryness, 16.274 g of dry residue was obtained, which made it possible to determine the extraction yield to be 16.3 per cent.

### 2.3. Film Preparation

Films were prepared using 1% (*w*/*w*) solutions of gelatin and sodium alginate at a 2:1 weight ratio. Distilled water containing 1% (*w*/*w*) glycerol was used as the base of the film-forming solution. A 50% ethanol extract of *P. sidoides* was incorporated at concentrations ranging from 0.25 to 1.0% (*w*/*w*). The resulting mixture was stirred at 600 rpm for 50 min at 90 °C until a homogeneous solution was obtained. The solution was then cooled to 50 °C. After cooling, transglutaminase (TGase) was added at 0.17% (*w*/*w*), and enzymatic cross-linking of the gelatin component was carried out at 50 °C for 80 min. The pH of the mixture was not adjusted during preparation. Calcium chloride (CaCl_2_) was added at a fixed concentration of 1.0% (*w*/*w*) to promote ionic cross-linking of the alginate component. The resulting film-forming solution was cast into molds using 80 mL per mold. The molds measured 12 × 12 cm, corresponding to a surface area of 144 cm^2^. The films were dried at 22 °C for 48 h, producing thin and uniform films [18,19,20]. After drying, the films were stored at 22 °C for 5 days before further analyses. Samples without the extract served as controls. The samples were labeled according to extract concentration: without CaCl_2_—0F, 0.25F, 0.5F, and 1.0F; with CaCl_2_—0Ca^2+^F, 0.25Ca^2+^F, 0.5Ca^2+^F, and 1.0Ca^2+^F.

### 2.4. Film Characterization

#### 2.4.1. Fourier-Transform Infrared Spectroscopy (FTIR)

The chemical structure of the samples was examined by Fourier-transform infrared spectroscopy (FTIR) using a Nicolet iS50 spectrometer (Thermo Scientific, Waltham, MA, USA). Spectra were recorded under controlled experimental conditions, with background correction performed before each measurement. The acquired spectra were subsequently evaluated to identify characteristic absorption bands associated with functional groups and molecular interactions within the materials.

#### 2.4.2. Mechanical Properties

The mechanical properties of the films were evaluated using a Zwick universal testing machine (model BDO-FBO 0.5TH) in accordance with ASTM D882 [21]. Prior to testing, the films were conditioned under laboratory conditions for 48 h. Measurements were conducted at a crosshead speed of 100 mm/min, and tensile strength and elongation at break were calculated from the resulting stress–strain curves.

#### 2.4.3. Water Vapor Transmission Rate (WVTR)

The water vapor transmission rate (WVTR) was determined in accordance with ISO 2528:2017 [22]. Film specimens were mounted over water-filled measuring vessels and sealed before being placed in a desiccator. WVTR was calculated using the following equation:

WVTR = (m × 24)/A

where WVTR is the water vapor transmission rate (g/m^2^ per 24 h), m is the mass increase (g), and A is the area available for water vapor transmission (m^2^).

#### 2.4.4. Swelling Index (SI_60_)

The swelling properties of the films were assessed after 60 min of immersion in distilled water. Before testing, film specimens measuring 3 cm × 3 cm were conditioned at ambient temperature and relative humidity for at least 24 h. Each specimen was then accurately weighed to determine its initial dry mass (W_0_).

The samples were immersed in 50 mL of distilled water at 25 ± 1 °C for 60 min. Following immersion, the specimens were retrieved, and residual surface water was carefully blotted off with absorbent paper. The hydrated films were immediately weighed to determine their mass after swelling (W_60_). The swelling index after 60 min (SI_60_) was calculated according to the following equation:

SI_60_ (%) = 100 × (W60 − W0)/W0

All measurements were conducted in triplicate, and the results were reported as mean ± standard deviation.

#### 2.4.5. Film Density (ρ_S_)

Film density was determined gravimetrically from the mass and calculated volume of individual specimens. Square samples measuring 3 cm × 3 cm were prepared from each film and conditioned at 23 ± 2 °C and 50 ± 5% relative humidity for at least 24 h before analysis.

The length and width of each specimen were measured using a digital caliper with an accuracy of ±0.01 mm. Thickness was measured at five locations per specimen using a digital micrometer with an accuracy of ±0.001 mm, and the mean thickness was used to calculate the sample volume (V) as the product of length, width, and thickness.

Each specimen was weighed on an analytical balance with a precision of ±0.1 mg. Film density was calculated using the following equation:

ρ_s_ = m/V

where ρ_s_ is the film density (g·cm^−3^), m is the specimen mass (g), and V is its volume (cm^3^).

At least five specimens were evaluated for each film formulation, and the results were presented as mean ± standard deviation.

#### 2.4.6. Film Colour Measurements

Film colour was evaluated using an EnviSense NR60CP colorimeter (Shenzhen ThreeNH Technology Co., Shenzhen, China) with an 8°/d measurement geometry. Colour was expressed using the CIELAB colour space recommended by the Commission Internationale de l’Éclairage (CIE).

The measured parameters comprised lightness (L*) and the chromatic coordinates a* and b*. The L* value ranges from 0, corresponding to black, to 100, corresponding to white. Positive a* values indicate red tones, whereas negative values indicate green tones. Similarly, positive b* values represent yellow tones, while negative values represent blue tones. Together, these coordinates provide a quantitative description of film colour and allow colour characteristics to be compared between samples.

#### 2.4.7. Surface Morphology and Elemental Composition

The surface morphology and elemental composition of the films were examined by scanning electron microscopy (SEM) coupled with energy-dispersive X-ray spectroscopy (EDX). Measurements were carried out using a Zeiss EVO 10 scanning electron microscope equipped with an EDX detector.

#### 2.4.8. Changes in Ionic Conductivity Following Film Immersion in Demineralized Water

Changes in ionic conductivity following film immersion in demineralized water were monitored using a multiparameter meter (TDS/EC/pH&TEMP-686). Film specimens measuring 3 cm × 3 cm were immersed in 100 mL of demineralized water. Conductivity and solution temperature were recorded at 0.5, 1.5, 3.0, 5.0, 10.0, 20.0, 25.0, 30.0, 40.0, 50.0, and 60.0 min after immersion to monitor changes associated with ion release [23].

#### 2.4.9. Antioxidant Activity Determined Using the DPPH and ABTS Radical Scavenging Assay

##### Preparation of Film Samples for Antioxidant Assays

Approximately 1 g of finely cut film was accurately weighed into a beaker and mixed with 10 mL of demineralized water. The mixture was maintained at 45 °C under vigorous magnetic stirring for approximately 1.5 h to promote dissolution and extraction of water-soluble constituents. The volume was subsequently adjusted to 10 mL with demineralized water, and the preparation was centrifuged to remove insoluble residues. The supernatant was collected and used as the stock solution for preparing serial dilutions for the DPPH and ABTS assays. The nominal stock concentration was approximately 100 mg film equivalents/mL and was calculated for each sample by dividing the accurately weighed initial film mass by the final preparation volume. Concentrations were expressed on an initial-film-mass basis rather than as the mass of dissolved solids recovered after centrifugation.

Antioxidant activity was assessed using the DPPH (2,2-diphenyl-1-picrylhydrazyl) radical scavenging assay according to [24]. The reduction in DPPH absorbance was measured spectrophotometrically at 517 nm using plastic cuvettes with an optical path length of 1 cm.

For each determination, 0.2 mL of the test sample was combined with 1.4 mL of DPPH solution in a 2 mL Eppendorf tube. The mixture was thoroughly mixed and incubated in the dark at room temperature for 30 min before absorbance was recorded. All samples were analyzed in five replicates.

DPPH radical scavenging activity was calculated using the following equation:

AA (%) = [(A_0_ − A_i_)/A_0_] × 100

where AA is the antioxidant activity (%), A_0_ is the mean absorbance of the DPPH solution, and A_i_ is the mean absorbance of the test mixture containing the sample and DPPH.

A mixture of 0.2 mL of demineralized water and 1.4 mL of methanol served as the spectrophotometric blank.

Antioxidant activity was also evaluated using the ABTS radical cation scavenging assay according to Re et al. [25].

A 0.10 mL aliquot of the test extract was combined with 1.90 mL of freshly prepared ABTS•^+^ working solution in a 2 mL Eppendorf tube. After thorough mixing, the reaction mixture was incubated in the dark at room temperature for 10 min. Absorbance was then measured at 734 nm using cuvettes with an optical path length of 1 cm.

The control was prepared by replacing the extract with 0.10 mL of the extraction solvent and adding 1.90 mL of ABTS•^+^ working solution. The corresponding solvent served as the spectrophotometric blank.

ABTS radical scavenging activity was calculated using the equation given for the DPPH assay, with A_0_ representing the mean absorbance of the ABTS•^+^ control and A_i_ the mean absorbance of the reaction mixture containing the test extract.

Serial dilutions of the film stock solutions were prepared and analyzed separately using the DPPH and ABTS assays. IC_50_ values were determined by linear regression of radical-scavenging activity (%) against film-equivalent concentration within the concentration range encompassing 50% scavenging. The relationship was expressed as y = ax + b, where y is radical-scavenging activity (%), x is the film-equivalent concentration, a is the slope, and b is the intercept. IC_50_, defined as the concentration required to achieve 50% radical scavenging relative to the radical control without the sample, was calculated as IC_50_ = (50 − b)/a. Concentrations were expressed as mg initial-film equivalents/mL rather than as the mass of dissolved solids.

#### 2.4.10. Optical Properties, Including Transmittance, Transparency, and Haze

Optical properties, including transmittance, transparency, and haze, were evaluated using a BYK-Gardner Haze-Gard I instrument (BYK-Gardner GmbH, Geretsried, Germany).

### 2.5. Fruit Storage and Analysis

The study used red, seedless grapes of the “Flame Seedless” variety from Namibia. Fruits were packaged in groups of four using 12 × 12 cm film sheets and then heat-sealed with a jaw sealer (FKR 200/12, Kegel Machines, Venray, The Netherlands). Three parallel replicates were prepared for each film type. Packaged fruits were placed in a chamber equipped with uniform illumination (Nature, 895 mm/45 W, High Lite, JUWEL Aquarium, Rotenburg an der Wümme, Germany) to ensure even lighting of the samples. Both the fruit wrapped in the tested films and the unwrapped fruit were exposed to light within the chamber throughout the incubation period. Conditions inside the chamber were monitored using a thermohygrometer (Figure 1). Anthocyanin content in the stored fruits was measured. Additionally, the effect of storage on the properties of the films was evaluated.

Total anthocyanin content in homogenized redcurrant fruits was determined spectrophotometrically. Samples were accurately weighed (±0.0001 g) and combined with 10 mL of methanol (equivalent to 7.7645 g). The mixtures were thoroughly mixed and left for 24 h to allow extraction, after which they were filtered to obtain clear extracts.

A 0.5 mL aliquot of each filtrate was transferred to a 2 mL Eppendorf tube and mixed with 1.5 mL of 0.1% (*v*/*v*) hydrochloric acid in methanol. Absorbance was measured at 528 nm against a reference blank consisting of 0.1% (*v*/*v*) hydrochloric acid in methanol.

Total anthocyanin content, expressed as cyanidin-3-O-glucoside chloride equivalents, was calculated as follows:

Total anthocyanins = (A × W)/(718 × m)

where A is the absorbance measured at 528 nm, W is the dilution/conversion factor accounting for all sample dilution steps, m is the mass of plant material used for extraction (g), and 718 is the specific absorbance of cyanidin-3-O-glucoside chloride at 528 nm.

### 2.6. Statistical Analysis

Independent measurements of technical parameters—such as mechanical properties (tensile strength and elongation at break), water vapor transmission rate (WVTR), swelling index (SI_60_), and density (ρ_s_)—were performed in triplicate, whereas optical properties (including transmittance, transparency, haze, and color measurements [L, a, b]) and antioxidant activity (determined using DPPH and ABTS radical scavenging assays) were measured in five replicates.

Biological analyses of total anthocyanin content in grape berries were performed in triplicate. The results are presented as the mean ± standard deviation. Statistical differences among treatments were evaluated using two-way and three-way ANOVA analysis of variance (ANOVA) followed by Tukey’s post hoc test. Differences were considered statistically significant at *p* < 0.05. Statistical analyses were performed using Statistica 13.3 software (TIBCO Software Inc., Palo Alto, CA, USA).

## 3. Results and Discussion

### 3.1. FTIR Analysis of Films

FTIR spectra (Figure 2) were used to assess the effects of extract concentration and storage time (0, 7, and 14 days) on the structure of films 0F, 0.25F, 0.5F, 1.0F, 0Ca^2+^F, 0.25Ca^2+^F, 0.5Ca^2+^F, and 1.0Ca^2+^F. Characteristic gelatin bands were observed in all samples. The broad band at 3300–3400 cm^−1^ was assigned to stretching vibrations of hydroxyl and amine groups, indicating the presence of an extensive hydrogen-bond network [26]. The band at 1645–1655 cm^−1^ corresponds to C=O stretching vibrations in the peptide structure (Amide I), while the band at approximately 1535–1550 cm^−1^ is associated with N–H bending and C–N stretching vibrations (Amide II). The band at 1230–1240 cm^−1^ further confirmed the presence of the protein component in the matrix. For alginate, characteristic bands appeared at 1600–1620 cm^−1^ and 1410–1430 cm^−1^, corresponding to asymmetric and symmetric stretching vibrations of carboxylate groups, respectively. Bands in the 1000–1100 cm^−1^ region were attributed to C–O–C stretching vibrations in the polysaccharide backbone [27].

Increasing extract concentration resulted in a gradual broadening and increase in the intensity of the 3200–3500 cm^−1^ band, which may indicate enhanced hydrogen-bonding interactions between phenolic compounds present in the extract and the hydroxyl and amine groups of the polymers. Additional changes were observed in the 1500–1600 cm^−1^ region, associated with aromatic ring skeletal vibrations, and in the 1200–1300 cm^−1^ region, corresponding to C–O stretching vibrations in phenolic structures. The observed changes in band position, intensity, and width indicate modifications in the chemical environment of the matrix and are consistent with the occurrence of non-covalent interactions, particularly hydrogen bonding, between compounds present in the extract and functional groups of the matrix components [28]. The most pronounced changes were observed at the 1.0% extract concentration, indicating a greater influence of the extract on the chemical environment of the polymer matrix. Due to the complex composition of *P. sidoides* extract, the observed spectral changes should be considered as the combined effect of its constituents interacting with the matrix.

Time-resolved spectral analysis revealed only minor changes in the intensity of the main bands after 7 and 14 days of storage, including slight narrowing of the hydroxyl/amine band and small shifts in the Amide I band. These changes may indicate gradual reorganization of the hydrogen-bond network and changes in the internal organization of the film structure. Overall, the FTIR results provide information on changes in the molecular environment within the protein–polysaccharide matrix, as reflected by changes in band intensity and position. The observed spectral changes are consistent with interactions among the protein, polysaccharide, and extract components.

Based on the FTIR results and the applied crosslinking methods, a conceptual model of the possible organization of the dual-crosslinked protein–polysaccharide matrix was proposed. The model illustrates possible interactions among the matrix components and provides a schematic representation of the structural organization of the films. A schematic representation of the proposed interactions is presented in Figure 3. This model should be regarded as a conceptual interpretation based on the observed spectral changes and the chemical properties of the matrix components.

### 3.2. Evaluation of Film Properties (SI60, ρ_S_, TS, EB, WVTR)

As the extract content in the studied films increased, an increase in film density was observed (particularly for film 1.0F, where ρs = 1.2004 ± 0.0151). The highest density was obtained for the sample containing Ca^2+^ alone without the addition of extract (0Ca^2+^F), indicating that ionic bridges between the carboxyl groups of the polysaccharide create a strongly cross-linked, compact structure. The addition of the extract to the Ca^2+^ system resulted in a slight decrease in density, suggesting partial reorganization of the ionic network. Analysis of the swelling index after 60 min (SI60) revealed clear differences between films with and without Ca^2+^, as well as depending on the extract content. In systems without Ca^2+^, high SI60 values were observed, which systematically decreased with increasing extract addition: from 7655 ± 70 for the control sample (0F) to 2628 ± 80 for the sample with the highest extract content (1.0F). In contrast, in the presence of Ca^2+^ ions, SI60 values were lower for all samples (e.g., 0Ca^2+^F = 5853 ± 49), indicating a reduced ability of the films to absorb water. The decrease in swelling in the presence of Ca^2+^ results from the formation of ionic bridges between guluronic acid residues in alginate (the “egg-box model”), which stabilize the structure and limit the mobility of polymer chains. Consequently, the space available for water is reduced and the film network becomes more compact. Additionally, the plant extract enhances these effects through hydrogen-bonding and hydrophobic interactions with polymer chains, which further limits swelling. These results confirm that the combination of enzymatic and ionic cross-linking allows the production of films with a more compact and hierarchically organized structure and reduced water absorption, which is beneficial for the application of the material as active films for fruit packaging. The swelling behavior largely depends on the extent of intermolecular interactions within polymer chains [29]. While cationic films swell in acidic environments due to protonation of amino and imine groups, anionic films swell in alkaline environments due to ionization of acidic groups [30,31,32].

Moderate swelling while maintaining flexibility was obtained in samples containing both Ca^2+^ and a moderate extract concentration (0.25Ca^2+^F–0.5Ca^2+^F).

The presence of ions and extract also clearly affected the mechanical strength of the obtained films. In mechanical tests for the films (Figure 4) without the addition of Ca^2+^ ions, the highest tensile strength was recorded for the control sample (0F: 20.41 ± 3.72 MPa), whereas increasing extract concentration led to a gradual decrease in strength to 15.14 ± 3.72 MPa at the highest concentration (1.0F). The introduction of Ca^2+^ ions significantly altered the mechanical behavior of the films. Similar results were obtained by He et al. [29], who observed that incorporating Toona sinensis extract into sodium alginate–gelatin films significantly reduced tensile strength from 455.73 MPa to 84.49 MPa. This substantial reduction can be attributed to the extract occupying intermolecular spaces between gelatin and sodium alginate, thereby potentially disrupting intermolecular interactions between these biopolymers and increasing the free volume and mobility of polymer chains within the film matrix [30]. A similar phenomenon was observed in sodium alginate–chitosan films containing essential oils [33], anthocyanins [34], and fig extracts [35].

In the present study, it was demonstrated that even without the addition of extract, ionic cross-linking increased the tensile strength to 25.06 ± 5.16 MPa (0Ca^2+^F), while simultaneously reducing elongation at break to 16.25 ± 12.31%. The increased tensile strength may be associated with enhanced cross-linking and intermolecular interactions within the film matrix [36]. A small addition of extract (0.25Ca^2+^F) further increased the tensile strength, reaching the highest tensile strength in the entire series (37.41 ± 2.45 MPa) while maintaining moderate elongation (20.22 ± 13.44%) [37]. Increasing the extract concentration above this level led to either a decrease in tensile strength or an increase in elongation, suggesting a possible partial disruption of the polymer network associated with the higher concentration of the functional component. Thus, dual cross-linking involving Ca^2+^ ionic interactions and enzymatic processes resulted in films with balanced mechanical properties, combining increased strength with retained flexibility. For comparison with commonly used packaging polymers, the 0.25Ca^2+^F film showed a relatively high tensile strength (37.41 ± 2.45 MPa). This value was higher than those reported for PBAT (20.1 ± 2.4 MPa) and PBSeT (14.7 ± 0.2 MPa), whereas its elongation at break (20.22 ± 13.44%) was substantially lower than that reported for PBAT (689.5 ± 110.3%) and PBSeT (917.6 ± 26.4%) [38,39]. LDPE films are also characterized by relatively high extensibility, with reported elongation at break values of at least 200%, although their mechanical properties depend strongly on film grade, thickness, orientation, and processing conditions [40]. Thus, the developed films combine relatively high tensile strength with limited extensibility, which can be attributed to the cross-linked polysaccharide network.

Although studies on protein–polysaccharide systems modified with plant extracts and cross-linked with Ca^2+^ are limited, the obtained results are consistent with known trends for biopolymers enriched with flavonoids [41,42]. To confirm the relationship between the degree of cross-linking and mechanical properties, microstructural analysis was performed using scanning electron microscopy (SEM) of both film surfaces and cross-sections. Both the 0.25Ca^2+^F and 1.0Ca^2+^F samples exhibited a dense, uniform, and compact structure without visible micropores, cracks, or signs of phase separation. Cross-sectional images revealed a homogeneous internal architecture, indicating good compatibility of the components and effective cross-linking of the matrix (Figure 5).

Elemental mapping (EDX) showed a relatively uniform distribution of calcium throughout the matrix of both samples, confirming the incorporation and homogeneous distribution of calcium-containing species within the polymer network (Figure 6). EDX analysis does not directly confirm the formation of ionic bridges. However, the distribution of calcium, together with changes in mechanical properties, swelling behavior, density, and film morphology, indicates the contribution of Ca^2+^ ions to the structure of the alginate–gelatin matrix. The presence of Ca^2+^ ions promotes ionic interactions with the carboxyl groups of alginate, contributing to the formation of a more compact and cohesive polymer network. The combined SEM and EDX observations indicate that the differences in mechanical strength between the samples are mainly related to the effect of extract concentration on the polymer network rather than to visible structural defects or phase separation. Similar results were reported by Li et al. [43], who observed that transglutaminase-catalyzed cross-linking of zein and ε-poly-L-lysine (ε-PL) created a compact polymer network structure, while the covalent coupling of hydrophobic ε-PL carbon chains with glutamine residues reduced the overall hydrophilicity of the film matrix.

The introduction of Ca^2+^ initially increased the WVTR to 66.5 ± 2.4 g/m^2^·24 h for 0Ca^2+^F and 71.8 ± 2.7 g/m^2^·24 h for 0.25Ca^2+^F. This increase may be associated with local changes in the organization and homogeneity of the polymer network induced by ionic interactions, which may affect the free volume and water vapor diffusion pathways without necessarily resulting in visible surface pores. At a moderate extract concentration, the WVTR decreased to 52.4 ± 1.6 g/m^2^·24 h for 0.5Ca^2+^F, while at the highest extract concentration it reached 49.8 ± 1.5 g/m^2^·24 h (1.0Ca^2+^F), suggesting a more compact organization of the polymer network associated with the presence of the plant-derived component. The matrix network formed by pectin and metal ions improved water resistance. The authors suggested that the presence of metal ions and phenolic compounds promotes cross-linking interactions between the polymer and the ions, resulting in reduced free volume and increased tortuosity of water vapor diffusion pathways. The observed relationship indicates that the effect of the extract on WVTR strongly depended on the presence of Ca^2+^. In systems without ionic cross-linking, the extract acted as a matrix-densifying agent, as indicated by the simultaneous decrease in WVTR and SI60 and the increase in density, suggesting a reduction in free volume and water vapor transport. In the presence of Ca^2+^, the initial increase in WVTR, despite the improvement in mechanical strength, indicates that the introduction of Ca^2+^ did not uniformly improve water vapor barrier performance and that ionic interactions may have modified the local organization of the polymer network and the pathways available for water vapor diffusion. At higher extract concentrations, the combined action of Ca^2+^ and the plant-derived component resulted in a more compact network organization, reflected in the simultaneous reduction in WVTR and swelling while maintaining favorable mechanical properties (0.25–0.5Ca^2+^F). These results suggest that the effect of Ca^2+^ on water vapor transmission depended on the overall composition of the films, and that the controlled combination of ionic interactions and extract incorporation enabled modulation of WVTR and mechanical properties.

### 3.3. Color Analysis of the Films and SEM Analysis

With increasing extract content, a systematic decrease in light transmittance was observed, both in systems without Ca^2+^ (from 92.41 ± 0.10% for 0F to 54.73 ± 0.53% for 1.0F) and in the presence of calcium ions (from 92.26 ± 0.10% for 0Ca^2+^F to 63.28 ± 0.29% for 1.0Ca^2+^F). Opacity is an important parameter for films intended for food packaging and/or coatings, as the visual appearance of the films can influence consumer acceptance depending on the application (Figure 7). Moreover, these parameters are also functional indicators of the size of particles dissolved in the polymer-forming solutions. Particles larger than visible wavelengths hinder light passage and increase membrane opacity [44]. Although transparent films that allow visualization of the packaged content are generally preferred by consumers, materials with active properties such as light barriers and reduction in oxidative reactions are increasingly sought due to their benefits for food stability. Plant extracts, rich in various active compounds, have the potential to interact with biopolymers, leading to diverse color changes [45,46,47]. This phenomenon was observed in the present study: an increase in the a* parameter (shift toward red) and a pronounced increase in b* (yellowness) were noted (Figure 8), especially at the highest extract concentrations in the 1.0F and 1.0Ca^2+^F samples. At the same time, lightness (L*) decreased (Figure 9) from approximately 92 to 54–60, indicating progressive darkening of the material. These changes corresponded with a simultaneous increase in haze, reaching 24.15 ± 1.03% for 1.0F and 23.65 ± 0.15% for 1.0Ca^2+^F, confirming increased light scattering in a more heterogeneous structure containing the extract. He et al. [31] similarly observed a decrease in L* from 88.88 ± 0.23 to 50.56 ± 0.65 with increasing concentrations of Toona sinensis extract in sodium alginate–gelatin films. In the present study, the presence of colored phenolic compounds in the Pelargonium extract led to light absorption in the visible range, directly causing reduced transparency and changes in a* and b* values. The introduction of Ca^2+^ modified these changes. The presence of Ca^2+^ alone did not significantly affect lightness but increased haze and slightly reduced transparency, indicating the formation of a microheterogeneous ionic network. At a moderate extract addition (0.25Ca^2+^F), relatively high transparency and moderate b* values were observed, suggesting a more homogeneous structural reorganization due to the synergy between ionic cross-linking and interactions with extract components. At higher extract concentrations (0.5–1.0Ca^2+^F), L* further decreased and b* increased, accompanied by enhanced light scattering. These results indicate that optical properties are closely linked to polymer network reorganization and the presence of colored extract components. The extract primarily affects light absorption (changes in L*, a*, and b*), whereas Ca^2+^ mainly influences light scattering through microstructural modification. Incorporating extracts containing polyphenolic compounds into biopolymer films typically increases opacity, as the presence of phases with different refractive indices reduces light transmission (Figure 7). These findings are consistent with previous studies by Adilah et al. [48], who incorporated mango peel extract into fish gelatin films and Rodsamrana & Sothornvita [49], who applied lime extract to pectin films. In contrast, Melo et al. [50] incorporated mango seed extract into starch-based films. In the present study, it was also evident that the appropriate selection of concentrations for both factors is crucial to obtain films with controlled transparency and color, which is highly important for designing active packaging materials for products requiring protection from light.

### 3.4. Evaluation of the Antioxidant Properties of the Films

The antioxidant activity of the developed protein–polysaccharide films was evaluated using DPPH and ABTS radical scavenging assays, with results expressed as IC_50_ values, i.e., the sample concentration required to neutralize 50% of free radicals (Figure 10). Lower IC_50_ values indicate higher antioxidant capacity of the material. The results confirmed the very high antioxidant activity of the 50% ethanol extract from *P. sidoides* root. The IC_50_ values for the extract alone were several times lower than those for all tested films, reflecting its high efficacy in radical scavenging. These properties are attributed to the presence of numerous phenolic compounds, such as phenolic acids, flavonoids, and coumarin derivatives, which can donate hydrogen atoms or electrons in reactions with reactive oxygen species. Films without extract (0F and 0Ca^2+^F), as well as those with very low extract content (0.25F and 0.25Ca^2+^F), did not show measurable antioxidant activity in the applied assays. This indicates that the protein–polysaccharide matrix alone does not possess significant antioxidant properties or that their levels remain below the detection limit of the DPPH and ABTS methods. With increasing extract content from 0.5% to 1%, a clear enhancement in film antioxidant activity was observed, manifested by decreasing IC_50_ values in both assays. This relationship confirms that the amount of incorporated extract directly affects the material’s radical-scavenging capacity. Higher extract content increases the number of available phenolic groups within the film structure capable of reacting with DPPH and ABTS radicals. Simultaneously, the antioxidant activity of the films was noticeably lower than that of the pure extract, which can be explained by the immobilization of bioactive compounds within the polymer matrix and their gradual release into the reaction medium. Protein–polysaccharide matrices can thus act as carriers for phenolic compounds, enabling controlled and sustained release from the material [51,52]. The ionic cross-linking with Ca^2+^ also had a significant influence on antioxidant activity. Films containing calcium ions exhibited higher IC_50_ values compared to the corresponding non-ionically cross-linked samples, indicating lower radical-scavenging efficiency. This effect can be explained by the formation of additional ionic bridges between the carboxyl groups of polysaccharides and Ca^2+^ ions, which densify and tighten the polymer structure. As a result, the diffusion of phenolic compounds from the matrix into the reaction medium is restricted, reducing their availability to react with radicals [53,54]. Increasing the *Pelargonium sidoides* extract content enhanced the radical-scavenging capacity of the films, as indicated by lower IC_50_ values in the DPPH and ABTS assays. Films containing Ca^2+^ exhibited higher IC_50_ values than the corresponding films without Ca^2+^, indicating lower measured antioxidant activity under the assay conditions. This difference may reflect changes in the accessibility of antioxidant constituents associated with polymer–polyphenol interactions and the organization of the crosslinked matrix. However, the present assays cannot establish the contribution of diffusion or identify the mechanism responsible for this difference.

The DPPH and ABTS results demonstrate antioxidant capacity under the experimental conditions but do not establish controlled or sustained release of bioactive compounds or prolonged antioxidant action during food storage. These properties require separate time-resolved release studies involving quantitative analysis of antioxidant constituents in suitable receiving media. The absence of such measurements is a limitation of the present study. Comparison of the results obtained with DPPH and ABTS assays showed that IC_50_ values in the ABTS test were slightly lower than those in the DPPH test. This may be due to differences in the nature of the radicals: the ABTS^+^• radical is more reactive and better soluble in aqueous environments, which facilitates its interaction with hydrophilic extract components and compounds released from the hydrophilic film matrix. In summary, the incorporation of *P. sidoides* root extract effectively imparts antioxidant properties to protein–polysaccharide films, with activity increasing alongside extract content. Meanwhile, Ca^2+^ cross-linking partially limits the immediate antioxidant activity of the material due to reduced availability of phenolic compounds in the densified polymer structure. However, this mechanism favors controlled, slow release of bioactive compounds, which is especially desirable in active food packaging systems, where prolonged protection against oxidative processes is critical.

### 3.5. Conductivity Changes in the Films

The conductivity analysis of the protein–polysaccharide films revealed a significant influence of water contact time, the presence of Ca^2+^ ions, and the concentration of *P. sidoides* extract on the matrix properties (Figure 11). Films without Ca^2+^ (0F–1.0F) initially exhibited low conductivity (24–27 µS), which systematically increased over 60 min to 67–91 µS. This increase indicates the gradual release of electrically conductive species from the matrix into the aqueous environment, which may include ions and polar components of the extract, consistent with previous observations in alginate-based biopolymer films [55]. The presence of Ca^2+^ ions (0Ca^2+^F–1.0Ca^2+^F) resulted in higher initial conductivity values upon water contact. This behavior may be associated with changes in ionic interactions within the alginate network and the release of ionic species upon hydration; however, conductivity measurements alone do not allow identification of the specific ionic species responsible for the observed changes. Increasing the extract concentration accelerated the rate of conductivity increase, suggesting a greater release of electrically conductive species from the matrix, potentially including ions and polar compounds derived from the extract. These results indicate that the incorporation of Ca^2+^ and the plant-derived extract affects the ionic transport behavior of the films, although the specific contribution of Ca^2+^ ions cannot be distinguished from that of other conductive species based on conductivity measurements alone. Overall, the studied films exhibited time-dependent changes in conductivity upon water contact, indicating the release of electrically conductive species while maintaining the structural integrity of the polymer matrix. Such behavior may be relevant to active food packaging applications, where controlled release of soluble components can contribute to the functional performance of the material [56,57,58,59].

### 3.6. Anthocyanin Content in Packaged Fruits

Anthocyanins are natural pigments present in fruits and vegetables and are widely distributed in nature. They differ in the number and position of hydroxyl and/or methoxy groups, as well as in the type, number, position, and acylation of the sugars present in their structure [60]. Their stability is influenced by factors such as temperature, light, oxygen, ascorbic acid, sulfur dioxide, the presence of enzymes (peroxidase, polyphenol oxidase, and glucosidase), metal ions, proteins, other flavonoids, as well as food processing and storage conditions [61]. In the studied films, which were hierarchically organized at the supramolecular level through enzymatic and ionic cross-linking, significant changes in anthocyanin content were observed during incubation, reflecting the dynamics of oxidative processes and stress responses in the stored fruits (Figure 12).

After 7 days of storage, a marked increase in total anthocyanin content was observed compared to day 0 (0NP: 0.079% ± 0.000), indicating activation of stress response mechanisms in the grapes. The highest levels were found in samples without active films (7F_NP: 0.160 ± 0.009) and in the variant with a film lacking plant extract (7F_0F: 0.153% ± 0.009). These results indicate that storage can induce oxidative stress and stimulate the accumulation or biosynthesis of phenolic compounds, including anthocyanins. This phenomenon has been widely described as an adaptive response of fruits to changes in temperature, oxygen availability, and other stress factors [62,63]. Anthocyanins act as natural antioxidants and may accumulate in response to increased oxidative stress. Variants containing plant extract exhibited moderately lower anthocyanin levels (7F_0.25F: 0.127% ± 0.006; 7F_0.5F: 0.133% ± 0.006; 7F_1.0F: 0.131% ± 0.005), which may indicate a lower intensity of stress-related anthocyanin accumulation in these fruits. However, anthocyanin content alone provides only indirect evidence of the oxidative status of the fruit and cannot be considered a direct measure of oxidative stress. Figure 13 shows images of the stored fruits previously packaged in the developed films. Phenolic compounds present in the extract may contribute to reducing oxidative stress by scavenging free radicals and chelating metal ions that catalyze oxidation reactions [64]. Consequently, the need for activation of the phenylpropanoid pathway responsible for anthocyanin biosynthesis may be reduced. Similar effects have been observed in active packaging materials containing plant extracts, which influenced the redox environment of stored fruits [49,65]. Films containing Ca^2+^ ions (7F_0Ca^2+^F: 0.158% ± 0.008) showed relatively high anthocyanin content, which may be associated with differences in the physiological response of the tissues during storage. In contrast, the highest concentration of Ca^2+^ combined with extract (7F_1.0Ca^2+^F: 0.112% ± 0.006) exhibited the lowest level among the 7-day samples. This may indicate an effect of ionic cross-linking and the presence of antioxidants, leading to an increased polymer network. According to polymer transport theory, increased cross-linking reduces free volume and limits gas diffusion through the matrix, directly affecting the rate of oxidative processes [66].

After 14 days of storage, the increase in anthocyanin levels was even more pronounced. The control sample without active film (14F_NP: 0.323% ± 0.068) had the highest value, reflecting a progressive stress response over the extended storage period. The variant with film lacking extract (14F_0F: 0.250% ± 0.027) showed lower values than the control, which may indicate an influence of the film on gas and moisture exchange. Hossen et al. reported that during room-temperature storage, anthocyanin levels initially increased and then sharply decreased at later stages [67,68,69]. They suggested that the transient increase might be related to gradual release from fruit cells in response to storage stress, particularly water deficit, while the subsequent decrease was attributed to natural degradation of anthocyanins by oxygen, oxidative enzymes, and tissue senescence. The changes observed in this study may be related to the functional properties of the films. Lower anthocyanin levels in systems containing extract and Ca^2+^ coincided with reduced WVTR, increased polymer network density, and restricted swelling. The denser structure may have limited moisture diffusion and modified the conditions affecting oxidative processes in the fruits. However, the observed differences in anthocyanin content should not be interpreted directly as evidence of improved anthocyanin preservation, since anthocyanin accumulation may itself represent a stress response of the fruit. The combination of enzymatic and ionic cross-linking with incorporation of plant extract allowed the development of a packaging system with integrated physical and chemical functionality.

## 4. Conclusions

Dual-crosslinked alginate–gelatin films containing *Pelargonium sidoides* extract were developed and characterized in terms of their functional properties. Ionic cross-linking with Ca^2+^ reduced swelling and increased tensile strength, while the addition of the extract affected the antioxidant activity of the films. An increase in extract content from 0.5% to 1% resulted in enhanced antioxidant activity, as indicated by decreased IC_50_ values in both the DPPH and ABTS assays. At the same time, the presence of Ca^2+^ reduced the measured antioxidant activity compared with the corresponding films without Ca^2+^. The combination of enzymatic and ionic cross-linking modified the mechanical properties, swelling behavior, and water vapor transmission, with the extent of these changes depending on the film composition. During grape storage, changes in anthocyanin content were observed depending on the film composition and storage time. After 7 days, the highest anthocyanin content was recorded in the control without a film (0.160 ± 0.009%), whereas in the variants containing the extract, the values ranged from 0.127 ± 0.006 to 0.133 ± 0.006%. After 14 days, the control reached 0.323 ± 0.068%. These differences indicate a varied response of the fruits during storage. The obtained results indicate that the application of Ca^2+^ and *Pelargonium sidoides* extract enables modification of the mechanical, physicochemical, water vapor transport, and antioxidant properties of the investigated films.

## Figures and Tables

**Figure 1:**
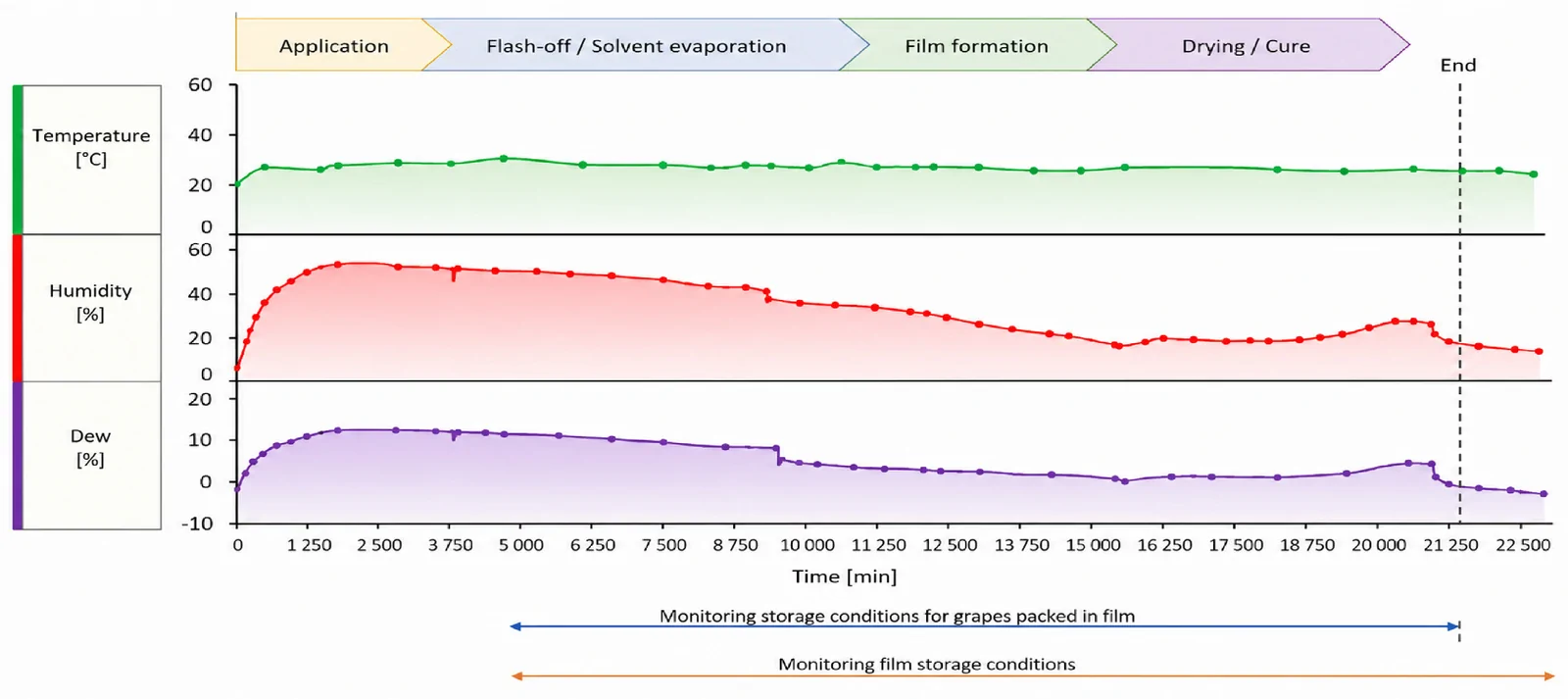
Monitoring of stored packaged grapes and films.

**Figure 2:**
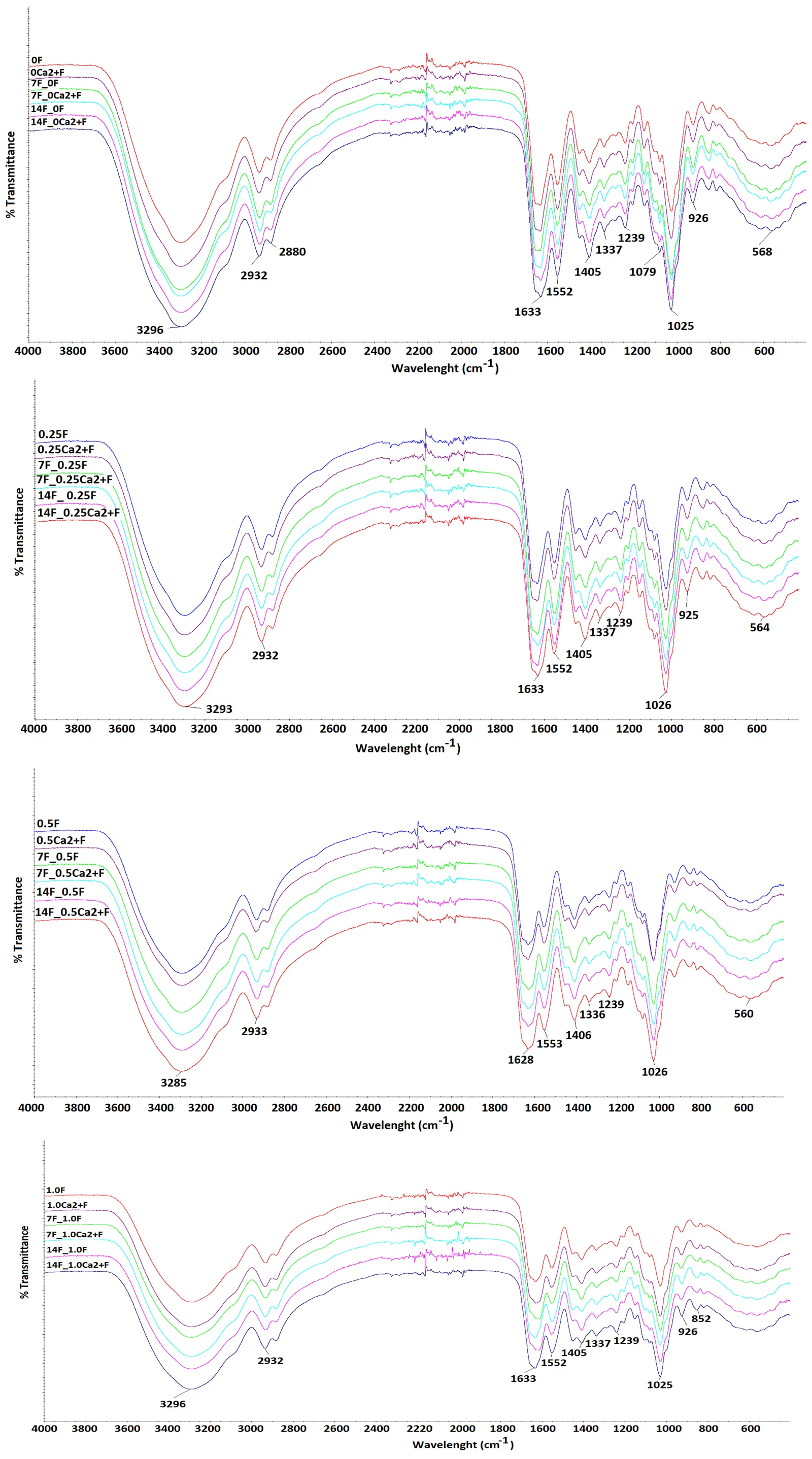
FTIR spectra of the obtained films during storage.

**Figure 3:**
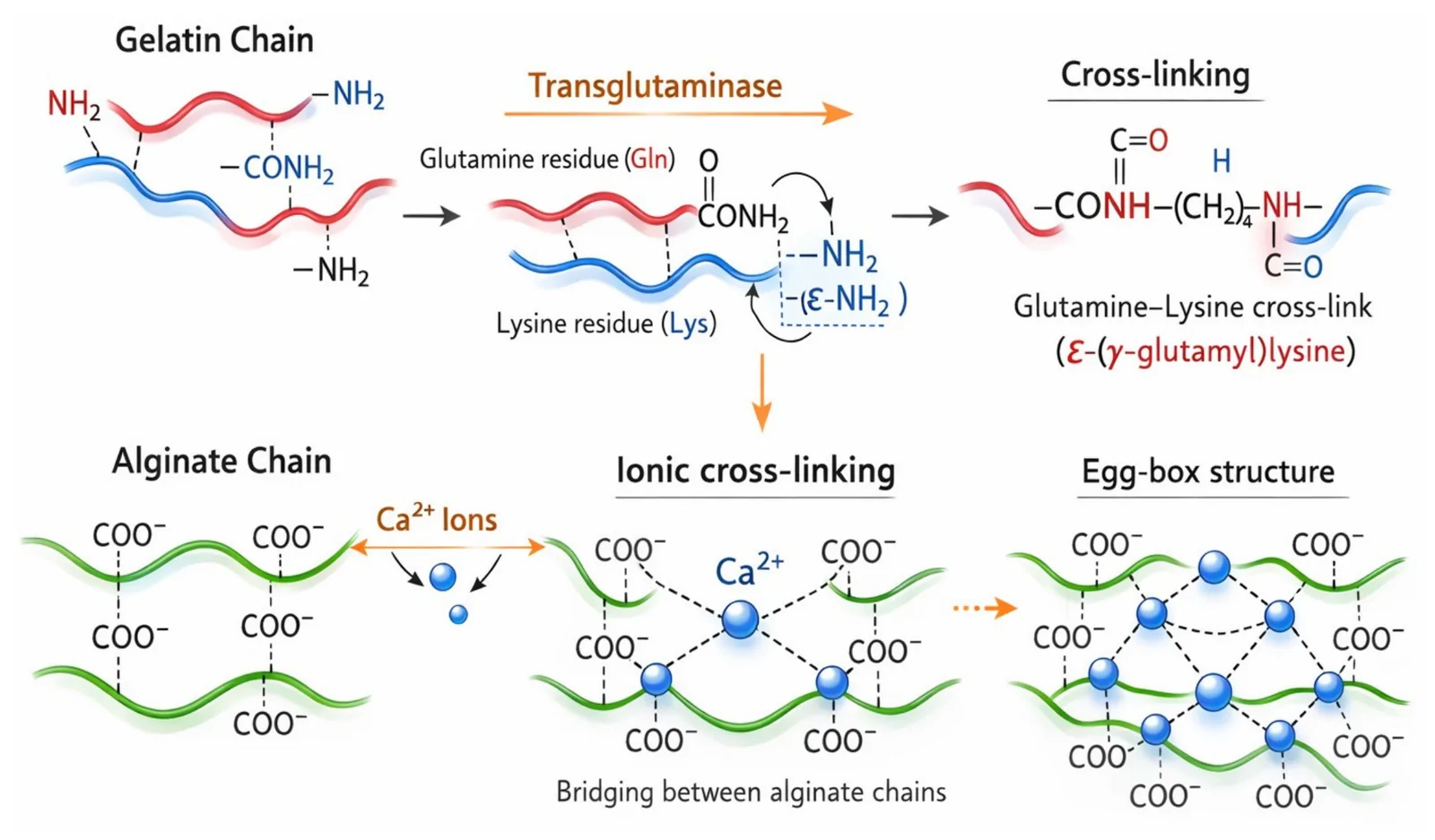
Mechanism of hierarchically organized supramolecular network formation in protein–polysaccharide films.

**Figure 4:**
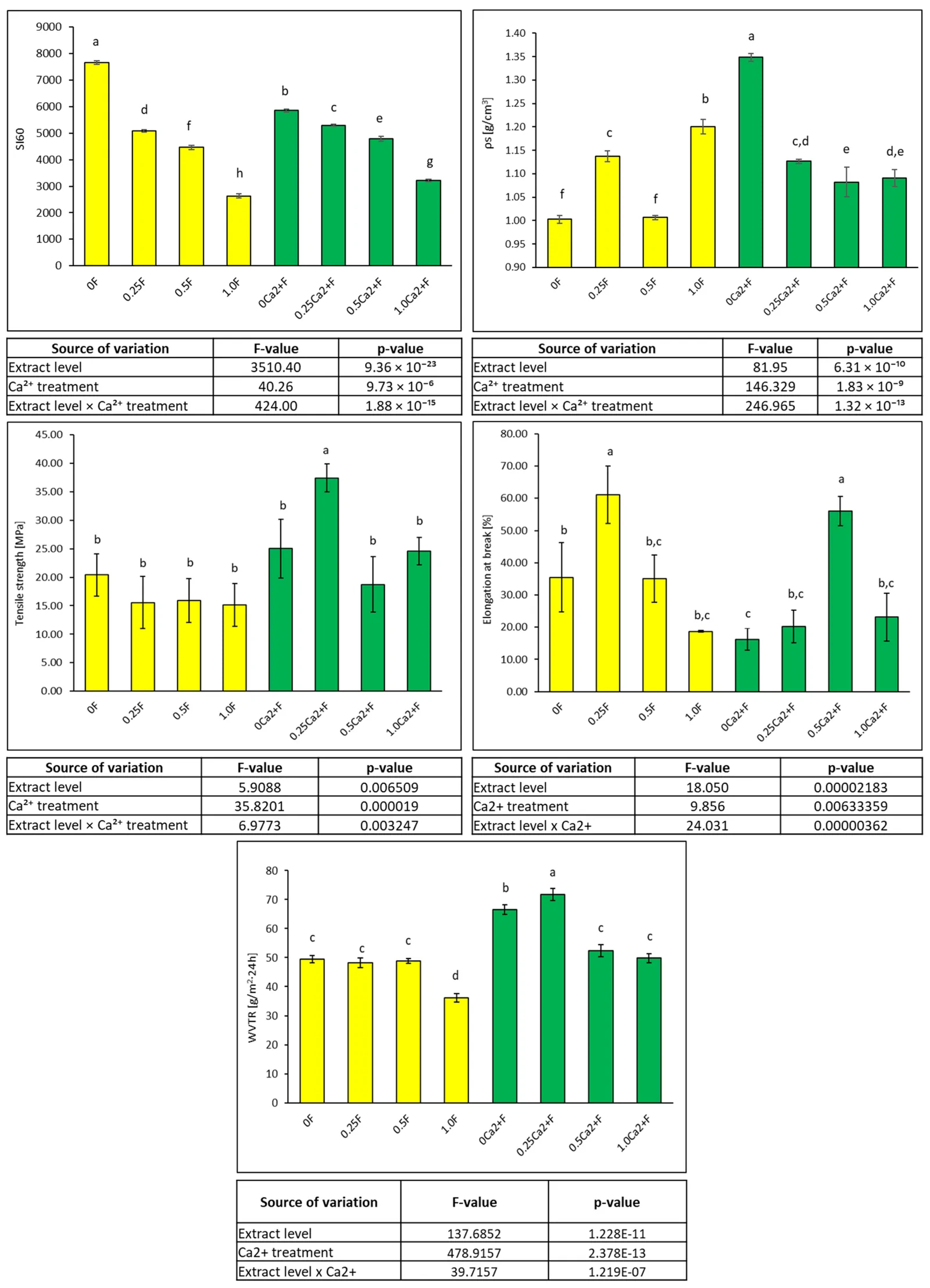
Film properties (SI60, ρs, TS, EB, WVTR) and determined parameters of two-way ANOVA with Tukey HSD test (α = 0.05).

**Figure 5:**
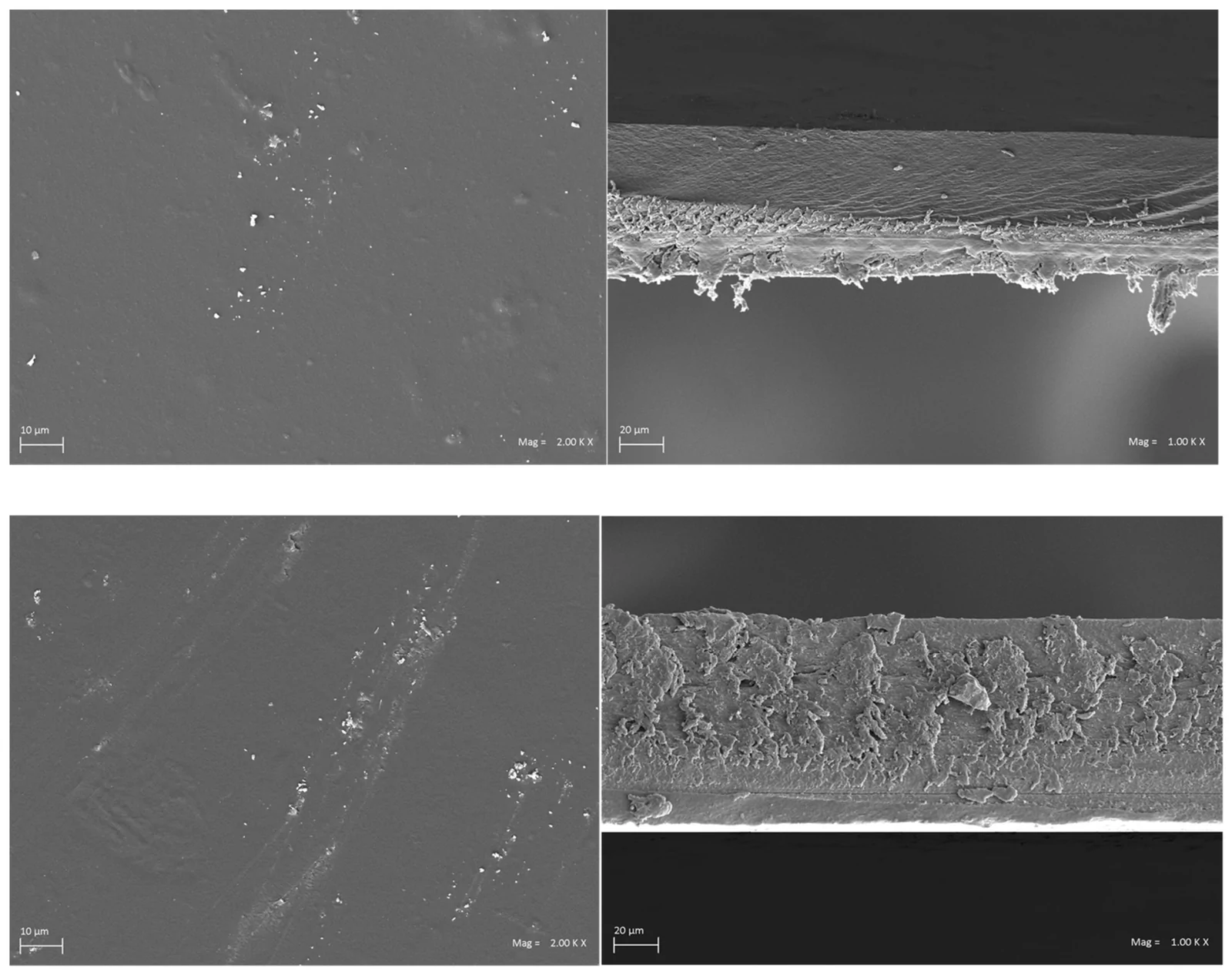
SEM images of the cross-sections of the 0.25Ca^2+^F and 1.0Ca^2+^F samples.

**Figure 6:**
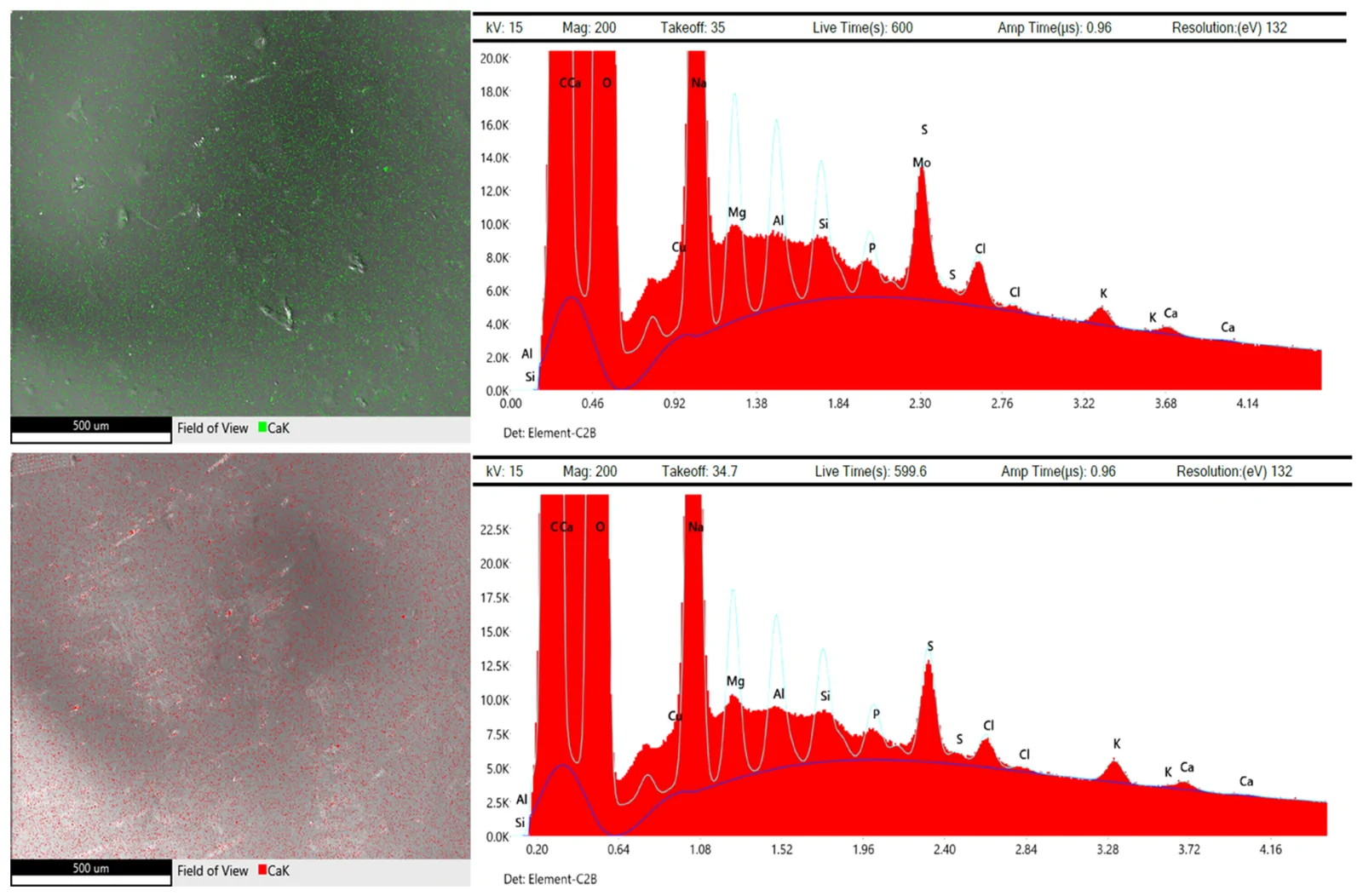
Mapping for samples: 0.25Ca^2+^F and 1.0Ca^2+^F with EDX.

**Figure 7:**
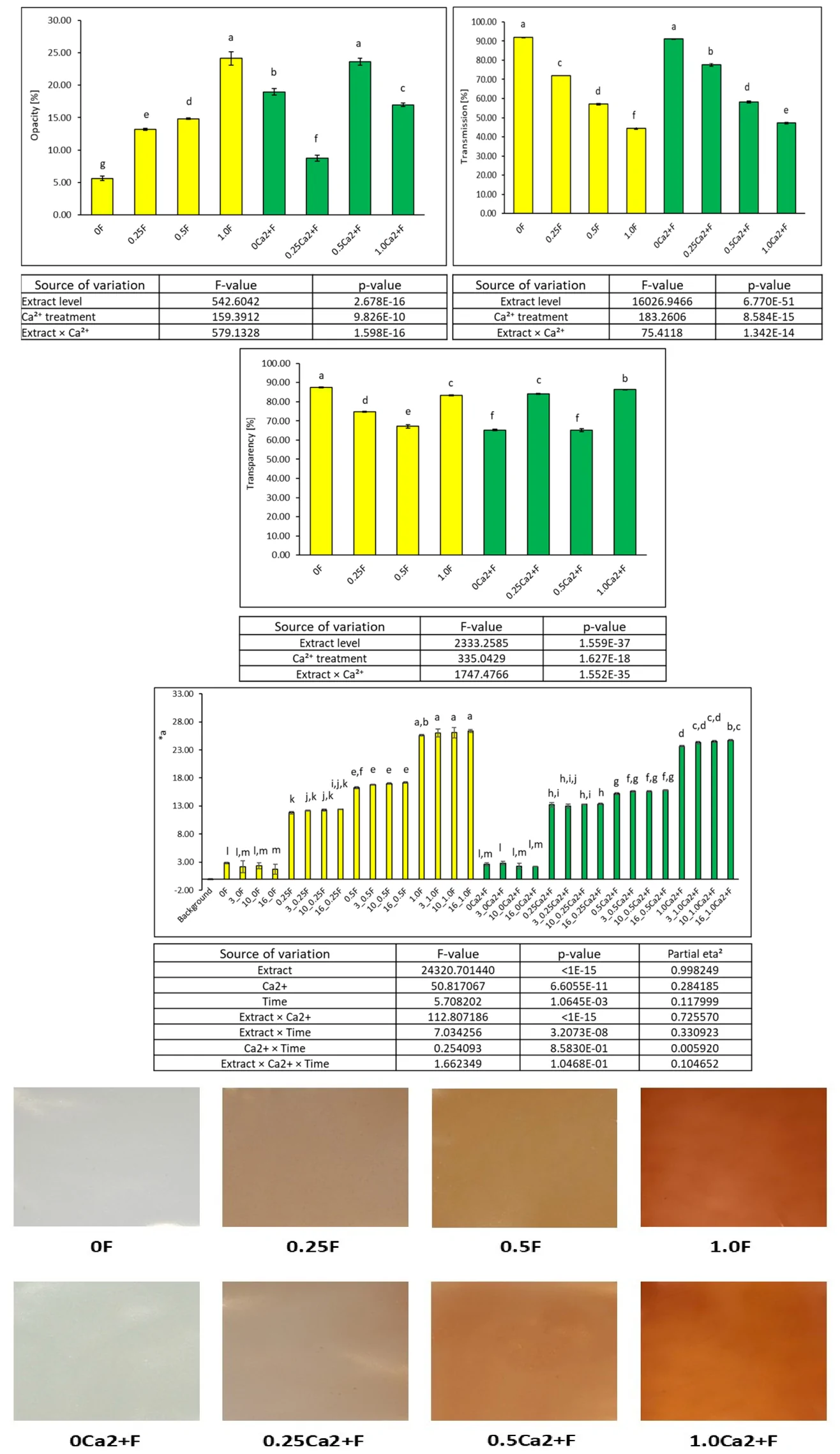
Optical and color properties of the films (opacity, transmittance, transparency, and a*) and determined parameters of two- and three-way ANOVA with Tukey HSD test (α = 0.05).

**Figure 8:**
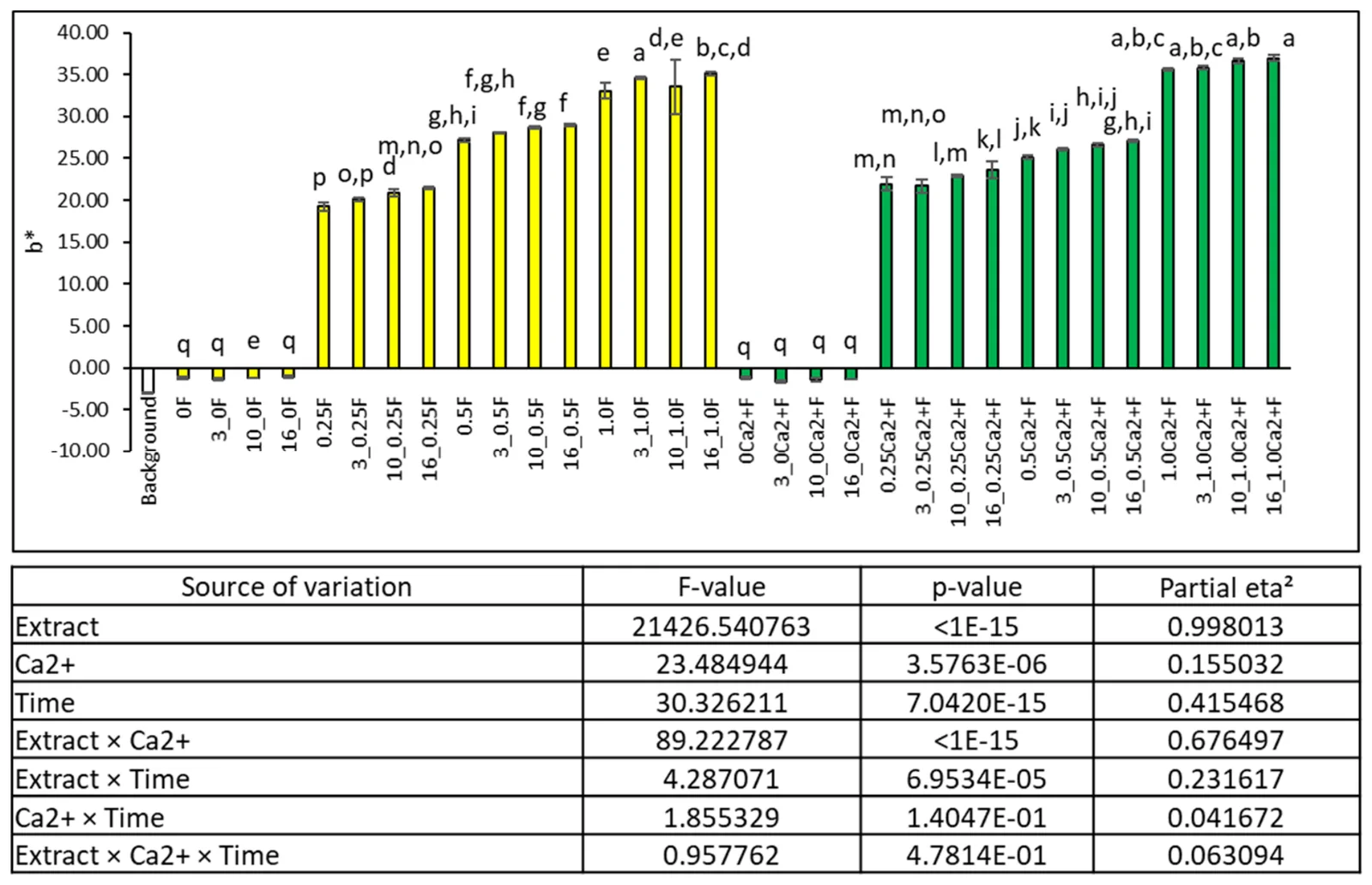
Results of the b* color parameter of the films and determined parameters of three-way ANOVA with Tukey HSD test (α = 0.05).

**Figure 9:**
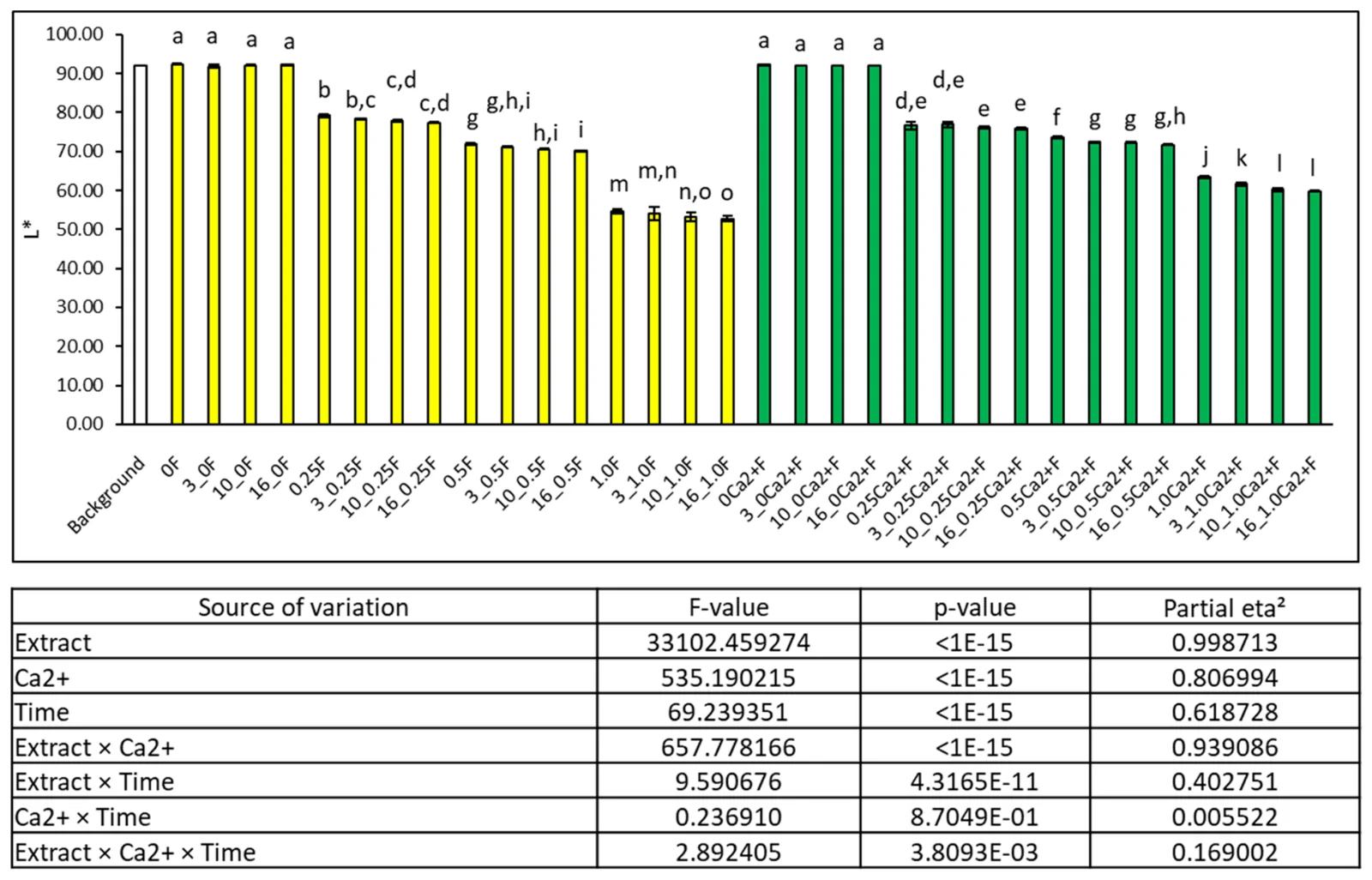
L* color parameter of the films and determined parameters of three-way ANOVA with Tukey HSD test (α = 0.05).

**Figure 10:**
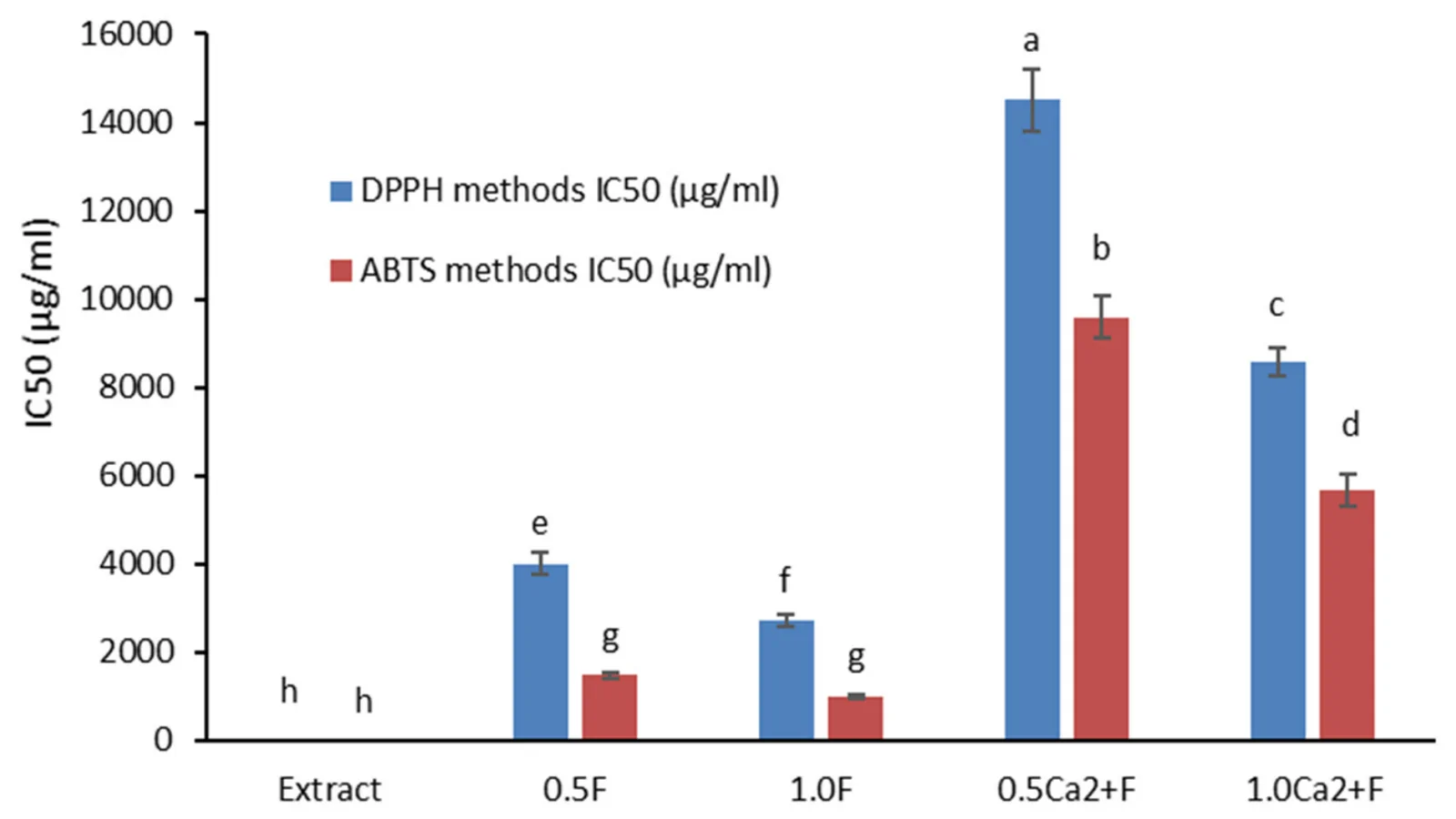
Antioxidant activity (DPPH and ABTS) of films and extract.

**Figure 11:**
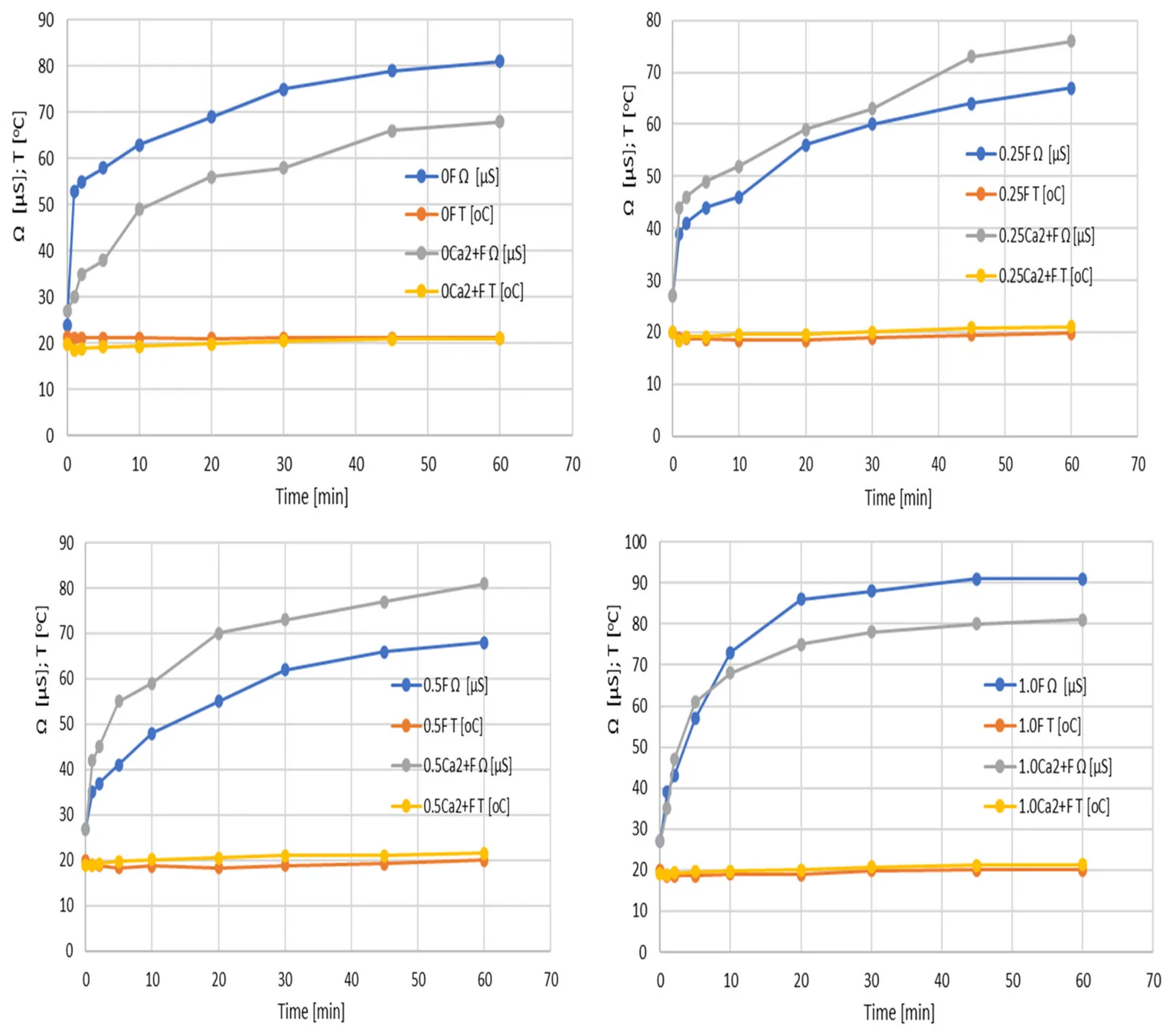
Conductivity results of the films.

**Figure 12:**
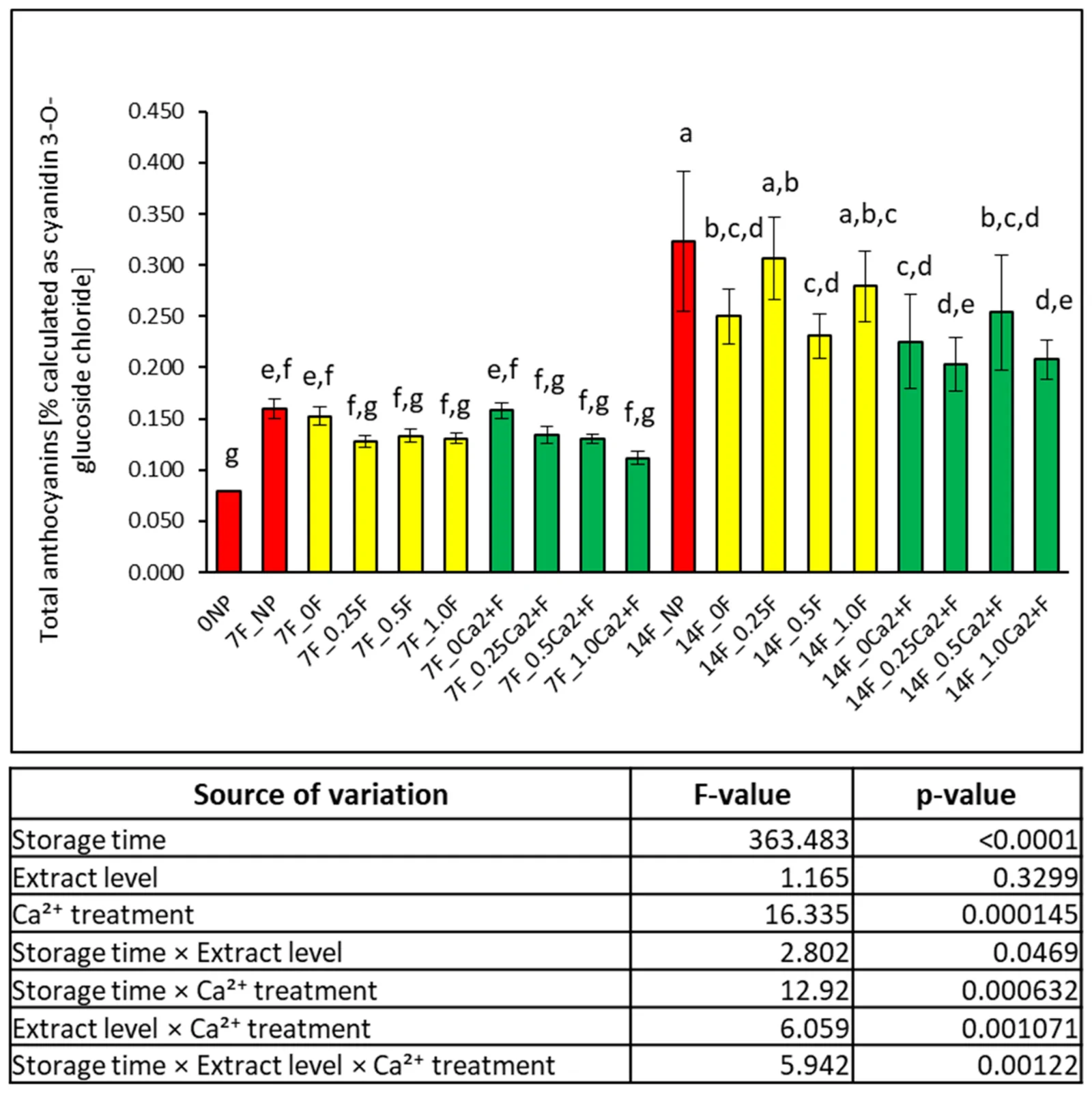
Results of anthocyanin content in packaged and unpackaged grapes and determined parameters of three-way ANOVA with Tukey HSD test (α = 0.05).

**Figure 13:**
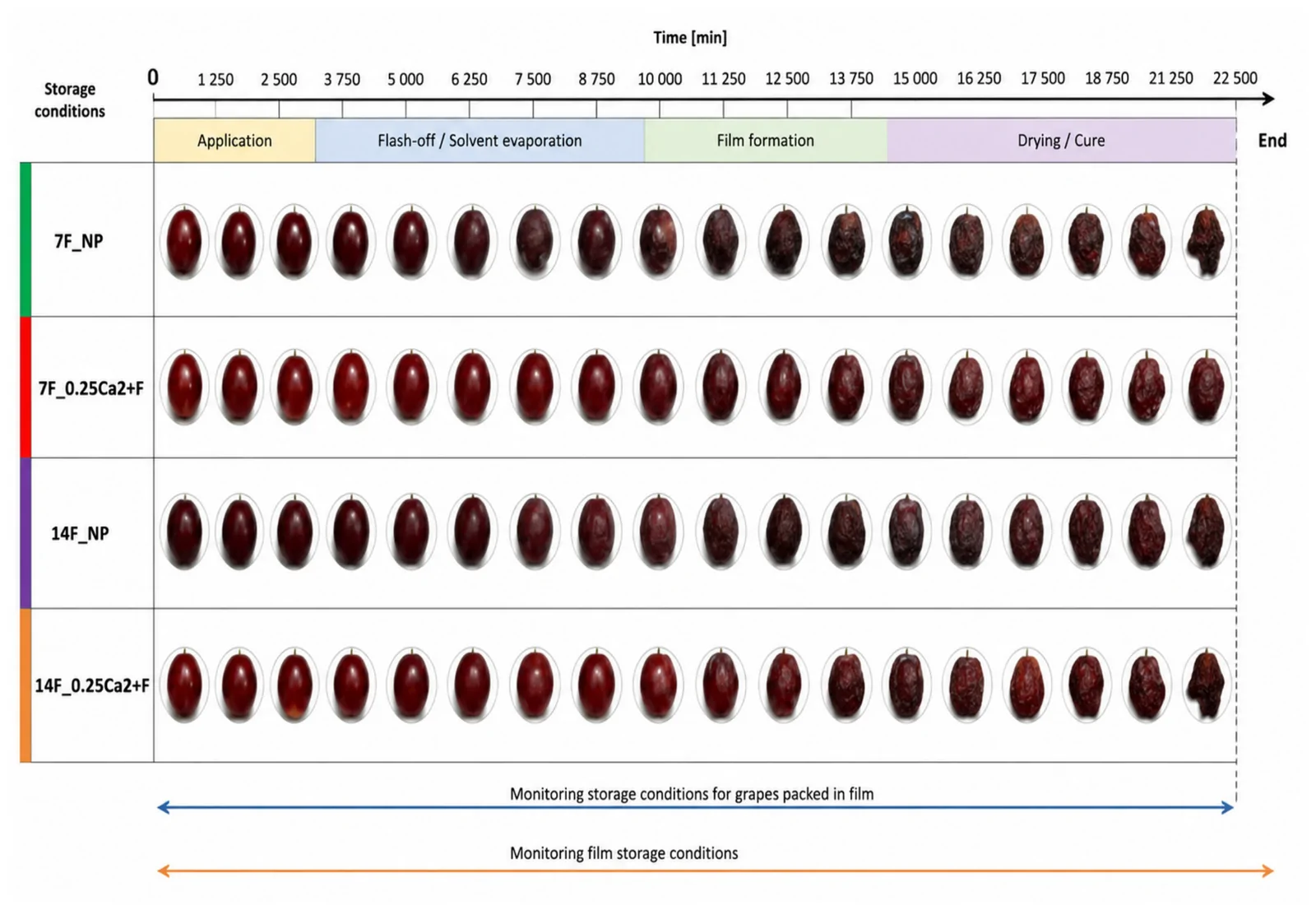
Images of fruits stored in the developed films.

## Data Availability

The original contributions presented in this study are included in the article. Further inquiries can be directed to the corresponding authors.
